# Granulocyte colony-stimulating factor treatment in women with premature ovarian insufficiency: a pilot clinical study of biological activity and menstrual resumption

**DOI:** 10.1186/s12958-025-01510-z

**Published:** 2026-01-07

**Authors:** Yasaman Sadeghi, Livia Deda, Mohammad Albar, Robert Casper

**Affiliations:** 1 IVIRMA Global Research Alliance, TRIO Fertility, Toronto, ON Canada; 2 Department of Obstetrics and Gynaecology, University of King Abdulaziz, Jeddah, Saudi Arabia; 3 Department of Obstetrics and Gynecology, University of Toronto, Toronto, ON Canada; 4 Lunenfeld-Tanenbaum Research Institute, Mount Sinai System, Toronto, ON Canada

**Keywords:** Primary ovarian insufficiency, Granulocyte Colony-Stimulating factor, Ovarian reserve, Follicle stimulating hormone, Menstruation, NCT06117982.

## Abstract

**Background:**

Women are born with a limited number of eggs, which decline over time. Premature ovarian insufficiency (POI) occurs when this decline happens before age 40, causing infertility. Bone marrow (BM) stem cells may help restore ovarian function, as some women conceive after BM transplants. Studies suggest that mobilizing stem cells with Granulocyte Colony-Stimulating Factor (G-CSF) can improve ovarian response in women with diminished ovarian reserve, possibly without needing ovarian infusion. Our study aimed to evaluate if G-CSF injections alone could improve ovarian function in women with POI.

**Methods:**

This was a pilot, non-randomized, open-label clinical trial including 11 women aged 25–40 years with clinical POI and menopausal symptoms, defined by elevated follicle-stimulating hormone (FSH) on two occasions, low anti-Müllerian hormone (AMH), and reduced antral follicle count (AFC). Participants received up to three rounds of subcutaneous G-CSF administered daily for four days per month over 60 days. Ovarian reserve markers (FSH, AMH, AFC), menstruation resumption, and menopausal symptoms were assessed at baseline and multiple follow-up points over 12 months.

**Results:**

The mean age of participants was 34.1 ± 5.2 years (BMI 23.96 ± 4.0 kg/m²). GCS-F injections resulted in significant increases in white blood cells and mild elevation of liver enzymes which returned to baseline within one month. By four months, significant improvements in menopausal symptoms were reported. Exploratory analyses did not identify consistent correlations between clinical response and baseline characteristics. Mean FSH decreased from 54.3 ± 24.6 IU/L at baseline to 29.0 ± 8.1 IU/L at six months (*p* = 0.008). AMH and AFC rose modestly (0.21 ± 0.15 to 0.49 ± 1.13 pmol/L; 1.09 ± 1.0 to 2.18 ± 2.60) but did not reach statistical significance. Menstruation resumed in 7 of 11 women (63.6%, *p* = 0.031). One participant showed marked response including retrieval of three mature oocytes.

**Conclusions:**

G-CSF injections were associated with menstrual resumption and symptom relief in most women with POI, suggesting biological activity. Although improvements in ovarian reserve markers were modest and disappointing in terms of the potential for assisted reproduction, these findings may support further evaluation of G-CSF in larger, controlled trials to clarify its clinical benefit and therapeutic potential.

**Trial registration:**

NCT06117982. https://clinicaltrials.gov/study/NCT06117982?cond=The%20Impact%20of%20Granulocyte%20Colony%20Stimulating%20Factor%20on%20Premature%20Ovarian%20Insufficiency&rank=1.

**Supplementary Information:**

The online version contains supplementary material available at 10.1186/s12958-025-01510-z.

## Introduction

It is generally accepted that women are born with all the eggs they will have in their lifetime, estimated to be about two million. There appears to be progressive depletion of oocytes such that by puberty, about four hundred thousand quiescent follicles are remaining, of which a small number initiate growth each month during a woman’s reproductive life. Depletion of this primordial pool of follicles occurs over time, resulting in menopause at around age 50 years in most women [1]. The rate at which the pool of resting follicles is lost may be accelerated because of genetic or chromosomal abnormalities (e.g. women with Turner’s syndrome or FMR-1 premutations), environmental factors (exposure to environmental toxicants or viral infection), iatrogenic causes (chemotherapy or radiation therapy for cancer) or in many cases from unknown causes [2]. Over time, women with accelerated loss of follicles may develop premature ovarian insufficiency (POI), also known as premature ovarian failure, defined by elevated gonadotropins, hypoestrogenism and anovulatory cycles below 40 years of age and impacting about 1% of women [3]. Women with POI develop ovarian resistance to gonadotropic signals, disrupting ovulation and menstrual cycles and ultimately rendering fertility treatments such as in vitro fertilization (IVF) ineffective. Currently, there are no effective non-invasive treatments to restore ovarian function in these women, highlighting a major clinical gap.

Notably, women who develop POI after chemotherapy have sometimes achieved spontaneous pregnancy following bone marrow (BM) transplantation, suggesting a potential regenerative role of bone marrow–derived stem cells (BMDSC) [4–6]. These stem cells may promote recruitment of pre-existing quiescent follicles through differentiation into oocyte-support cells (granulosa, theca, cumulus, or stromal cells) or by releasing paracrine and angiogenic factors that improve ovarian perfusion and follicular sensitivity to gonadotropins [7, 8].

There is increasing evidence to suggest that BMDSCscan restore ovarian function in both animal models and human studies [8–13]. This concept has led to clinical approaches in which hematopoietic stem cells are mobilized either through direct BM aspiration or, more recently, using granulocyte colony-stimulating factor (G-CSF) to move stem cells from the BM into peripheral circulation for potential therapeutic use. Traditionally, mobilized stem cells have been infused into the ovarian stroma or artery, procedures that are invasive and technically demanding.

In one study from Spain, women who had diminished ovarian response (DOR) and were considered as poor responders to gonadotropins were treated with G-CSF for 5 days at a mean dose of 600 mcg/d [9]. Peripheral blood was collected to isolate BMDSCs, and these stem cells were subsequently infused into one ovarian artery, using the other ovary as a control. The authors demonstrated that autologous stem cell ovarian transplantation (ASCOT) following G-CSF treatment resulted in improvements in antral follicle count (AFC) and anti-Müllerian hormone (AMH) levels in 81% of patients (13/16). Overall, comparing pre- and post-ASCOT IVF cycles, they noted improvements in AFC, a reduced cycle cancellation rate, and better fertilization rates. A total of five pregnancies occurred post-ASCOT treatment (three spontaneous) [9]. Interestingly, although only one ovarian artery was infused, an effect on the control ovary was also observed, suggesting that stem cells and/or their released factors may act systemically. This observation raised the question of whether direct ovarian infusion is necessary, or if mobilization of BMDSC into circulation alone might achieve similar effects.

As a result, the same group performed a follow-up study in 10 women with POI undergoing ASCOT compared to 10 women receiving only GCS-F (mean dose 600 mcg/d for 5 days) without apheresis and intraovarian artery infusion of hematopoietic stem cells [10]. Both groups yielded similar results, with six out of 10 women in each group exhibiting visible basal antral follicles. Additionally, two patients in each group underwent IVF, and one patient had a single embryo transferred in each group. This study suggests that intra-ovarian artery infusion of stem cells may not be required for folliculogenesis.

Based on these promising results in women with DOR, the objective of the present study was to determine if hematopoietic stem cell mobilization could be beneficial in improving ovarian response in women with POI. Since both bone marrow aspiration and ASCOT are invasive procedures, we chose to use G-CSF injections and stem cell mobilization from the BM alone, similar to the previous study in DOR women [10]. G-CSF treatment is safe and has been routinely used in healthy donors for decades to prepare them for allogeneic stem cell transplantation.

## Methods

### Study design and ethics

This was a pilot, non-randomized, open-label clinical trial evaluating the effect of G-CSF on ovarian reserve in women with POI. The study was reviewed and approved by the Veritas Independent Review Board. Additionally, Health Canada authorized the trial under Part C, Division 5 of the Food and Drug Regulations, with no objection to its initiation. The study is also registered on ClinicalTrials.gov (NCT06117982; Registration Date: 10/31/2023). All research procedures were conducted in accordance with the Declaration of Helsinki and applicable regulatory and institutional ethical standards.

### Eligibility criteria

Participants were women aged 25–40 years diagnosed with clinical POI and menopausal symptoms such as hot flashes, night sweats, insomnia, and vaginal dryness. The laboratory diagnosis of POI included two measurements of serum follicle-stimulating hormone (FSH) ≥ 30 IU/L at least one month apart, AFC < 5 and AMH < 3 pmol/L (0.43 ng/ml).

Exclusion criteria included autoimmune disorders, hematopoietic malignancies, sickle cell disease, or comorbidities that precluded infertility treatment or pregnancy (e.g., HIV/AIDS, hepatitis B/C, breast cancer, or Body Mass Index (BMI) > 40 kg/m²). Concurrent use of other medical or fertility treatments was not permitted, except for natural estrogen therapy for vasomotor symptom management. Women with idiopathic POI were primarily included; those with known autoimmune or genetic causes were excluded to minimize etiologic heterogeneity.

### Recruitment and consent

Eligible participants received both verbal and written study information and were given time to review the materials at home. Follow-up phone calls were made for those who agreed to be contacted. A total of 11 participants provided written informed consent and enrolled in the study.

### Intervention and clinical procedures

Participants attended an in-person baseline visit for assessment and medication administration. Pre-treatment assessments included hormonal testing (FSH, AMH, AFC), a complete blood count (CBC), liver enzymes ((Alanine Aminotransferase (ALT), Alkaline Phosphatase (ALP), Lactate Dehydrogenase (LDH)), renal function tests (creatinine, sodium, potassium, uric acid), and a spleen ultrasound.

Each participant received a 0.5 mL subcutaneous injection of 300 µg G-CSF (Neupogen^®^ prefilled syringes, Amgen, USA) for four consecutive days. The first injection was administered in the clinic and followed by a 60-minute observation period. Subsequent injections were self-administered at home after training. All baseline tests, except AMH and AFC, were repeated on the day after the fourth injection.

**Fig. 1:**
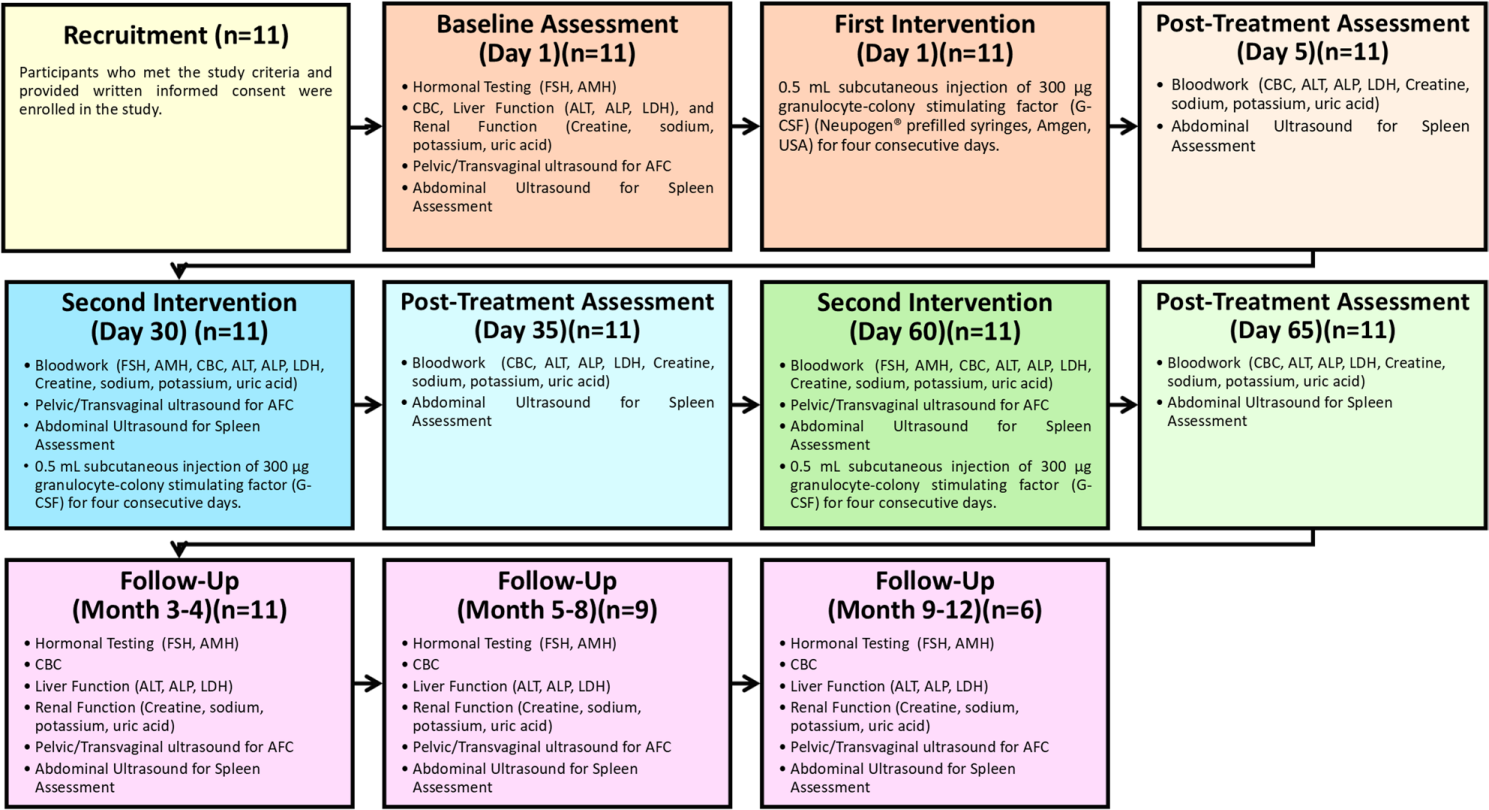
Flowchart illustrating the full study timeline, from enrollment through follow-up. The diagram details all interventions administered, the sequence of clinical visits, and the specific laboratory and imaging assessments performed at each scheduled time point

Participants returned on Day 30 for reassessment, including all baseline tests, and a second four-day G-CSF cycle, with follow-up testing on Day 35. A third four-day G-CSF cycle was offered at Day 60 if there was no measurable improvement in FSH, AMH or AFC. Monthly follow-up visits were conducted for up to 12 months. Patients with a reduction in FSH to < 20 IU/L and an increase in basal antral follicle count were offered IVF to assess the possibility of oocyte retrieval and embryo development. A participant flow diagram summarizing enrollment, follow-up, and analysis is provided for clarity (Fig. 1).

### Study endpoints

The primary endpoint was the change in serum FSH from baseline to month 4. Secondary endpoints included changes in AMH and AFC, and the resumption of spontaneous menstruation. The latter was a predefined exploratory outcome based on prior clinical observations.

### Statistical analysis

All analyses were performed using IBM SPSS Statistics, Version 29.0 (IBM Corp., Armonk, NY). Continuous variables were tested for normality using the Shapiro-Wilk test. Normally distributed variables were reported as mean ± standard deviation. Repeated-measures analysis of variance (ANOVA) was used to assess longitudinal changes in FSH, AMH, and AFC across three time points: baseline, month 4, month 6, and month 12. Where the overall F-test was significant, Bonferroni-corrected pairwise comparisons were conducted as post hoc tests. Changes in menstruation status (yes/no) between baseline and month 4 were analyzed using McNemar’s test.

Missing data were handled via listwise deletion. Given the small sample size and exploratory nature of the study, the analysis was considered underpowered for confirmatory inference; results should be interpreted as hypothesis-generating. Additionally, one participant who demonstrated an exceptional clinical response (including successful oocyte retrieval) was described separately in the results. Statistical significance was defined as a two-tailed *p*-value < 0.05.

## Results

### Patient demographics

A total of eleven patients diagnosed with POI were enrolled in the study, with the first participant enrolled on October 13, 2023, and the final participant recruited on June 13, 2024. The mean age at baseline was 34.1 ± 5.2 years, and the mean BMI was 23.96 ± 4.0 kg/m². At baseline, participants demonstrated severely diminished ovarian reserve, with mean FSH of 54.26 ± 24.65 IU/L, AMH of 0.12 ± 0.15 pmol/L, and AFC of 1.09 ± 1.04 follicles. Enrollment in the study was stopped after these 11 patients, as we observed no robust clinical response in AFC following G-CSF treatment. Instead, we decided to follow the initial 11 subjects for up to 12 months to determine if there was any long-term response.

Table 1 lists the demographic and clinical characteristics of the 11 patients included in this study with POI. In terms of reproductive history, seven participants were nulligravid, while four had a history of previous pregnancies (gravidity range: 0–3; parity range: 0–1). A family history of POI was reported by four patients (36.3%). The average duration since POI diagnosis was 4.18 ± 4.3 years.

**Table 1:** Demographic and clinical characteristics of individual patients

Case	Age at Baseline	z	BMI	Family History of POI	Duration of amenorrhea/POI Diagnosis (Years)	History of HRT	FSH ^a^ (IU/L)	FSH ^b^ (IU/L)	AMH ^a^ (pmol/L)	AMH ^b^ (pmol/L)	AFC ^a^	AFC ^b^	Menstruation ^a^	Menstruation ^b^
1	34.00		22.9	No	1.5	Yes	32.5	28.10	0.20	0.12	2.00	2.00	No	No
2	34.00		26.6	No	6	Yes	50.0	40.40	0.19	0.07	0.00	0.00	No	Yes
3	31.00		21.8	No	2.5	Yes	48.1	5.97	0.11	0.33	3.00	5.00	No	Yes
4	40.00		22.1	Yes	1	Yes	84.9	2.55	0.30	0.36	1.00	1.00	Yes	Yes
5	38.00		20.8	Yes	3	No	100.0	74.50	0.12	0.09	1.00	3.00	No	No
6	38.00		23.7	Yes	4	No	81.4	76.00	0.06	0.09	0.00	0.00	No	Yes
7	38.00		20.5	No	4	No	41.5	40.20	0.02	0.08	0.00	0.00	No	Yes
8	**25.00**		**21.3**	**No**	**6**	**Yes**	**30.0**	**6.38**	**0.55**	**3.89**	**1.00**	**8.00**	**No**	**Yes**
9	32.00		30.6	No	16	Yes	63.2	63.90	0.40	0.08	2.00	1.00	No	No
10	40.00		21.3	Yes	1	Yes	35.3	7.03	0.26	0.25	2.00	4.00	No	Yes
11	26.00		32.0	No	1	No	30.00	20.70	0.12	0.11	0.00	0.00	No	No

*BMI* Body Mass Index, *POI* Premature Ovarian Insufficiency, *HRT* Hormone Replacement Therapy, *FSH* Follicle Stimulating Factor, *AMH *Anti-Mullerian Hormone, *AFC *Antral Follicle Count

a: Values at Baseline; b: Values at the 4th month post treatment

Menopausal symptoms were assessed at baseline and again at month 4. At baseline, the most reported symptoms included hot flashes (6/11; 54.5%), irritability (5/11; 45.5%), poor concentration (5/11; 45.5%), vaginal dryness (7/11; 63.6%), insomnia (7/11; 63.6%), and night sweats (7/11; 63.6%). Less frequently reported symptoms included mood swings, weight gain, and memory difficulties.

At 4 months post-treatment, several symptom domains demonstrated improvement. The prevalence of hot flashes decreased to 2/11 (16.6%), although this reduction did not reach statistical significance (McNemar’s test, *p* = 0.125). Statistically significant improvements were observed in the prevalence of vaginal dryness (0/11; *p* = 0.016), insomnia (1/11; *p* = 0.031), and night sweats (1/11; *p* = 0.031). Although reductions in irritability and poor concentration were noted, these changes did not achieve statistical significance.

### Safety profile: Short-Term side effects and laboratory changes

The treatment was well tolerated, with no serious adverse events reported throughout the study. The most observed side effect was lower back pain (6 out of 11 participants (54.5%)) and mild to moderate headache, experienced by 5 out of 11 participants (45.4%) within 24 h following G-CSF administration. Other transient effects included fatigue (2 out of 11 participants (18.1%)) and injection site discomfort (9.0%). All symptoms were self-limited, resolving spontaneously within 1–2 days without the need for medical intervention.

Routine laboratory monitoring, including CBC, liver enzymes, renal function markers, and spleen size via ultrasound, was performed at baseline, 1 day following each G-CSF treatment course and monthly thereafter. Significant transient increases were observed in white blood cell (WBC) count a day after the final G-CSF injection. The mean WBC count at baseline was 5.6 ± 0.26 × 10⁹/L, which increased to 29.0 ± 4.3 × 10⁹/L on Day 5. Repeated-measures ANOVA revealed that this increase in WBC on Day 5 compared to baseline was significant (F (2, 22) = 30.94, *p* < 0.001). However, by Day 30, WBC levels returned to baseline values (mean 5.4 ± 0.37 × 10⁹/L), with no significant difference between Day 30 and baseline (*p* > 0.05), indicating that the increase was transient. This pattern of WBC increase was seen immediately after injections and returned to baseline after one month and was consistent across subsequent injections.

Regarding liver function tests, ALP and LDH showed a significant increase from baseline to Day 5. The mean ALP at baseline was 67.2 ± 5.6 U/L, which increased to 179.2 ± 19.4 U/L on Day 5 (*p* = 0.001). Similarly, LDH increased from a mean of 182.2 ± 5.7 U/L at baseline to 344.7 ± 26.2 U/L on Day 5 (*p* = 0.001). However, these increases were transient, as both ALP and LDH levels returned to baseline after one month (mean ALP: 72.44 ± 7.0 U/L, LDH: 185.8 ± 10.2 U/L), with no significant difference between Day 30 and baseline (*p* > 0.05). This trend was also observed after each subsequent treatment cycle, where ALP and LDH levels increased post-injection and returned to baseline after one month. Importantly, ALT levels remained stable throughout the study period, showing no significant changes over the treatment period.

Renal function was consistently normal throughout the study period. Tests for creatinine, sodium, potassium, and uric acid showed no significant changes from baseline to Day 30 or at any subsequent time points, confirming that no renal adverse effects were associated with G-CSF treatment. No evidence of clinically significant organ dysfunction, neutropenia or renal dysfunction. No splenomegaly was observed on ultrasound. All other laboratory markers remained stable throughout the study period.

### Hormonal and ovarian reserve changes

We assessed FSH, AMH, and AFC levels in patients over six months following the initial administration of G-CSF. Unfortunately, two of the eleven participants withdrew from the study after the third month.

### FSH levels

Regarding FSH levels over time, the mean FSH level decreased from a baseline of 54.26 ± 24.6 IU/L to 33.24 ± 28.01 IU/L four months post-treatment. A paired-samples t-test indicated a significant reduction in FSH levels from baseline to four months (t (9) = -2.511, *p* = 0.033).

**Fig. 2:**
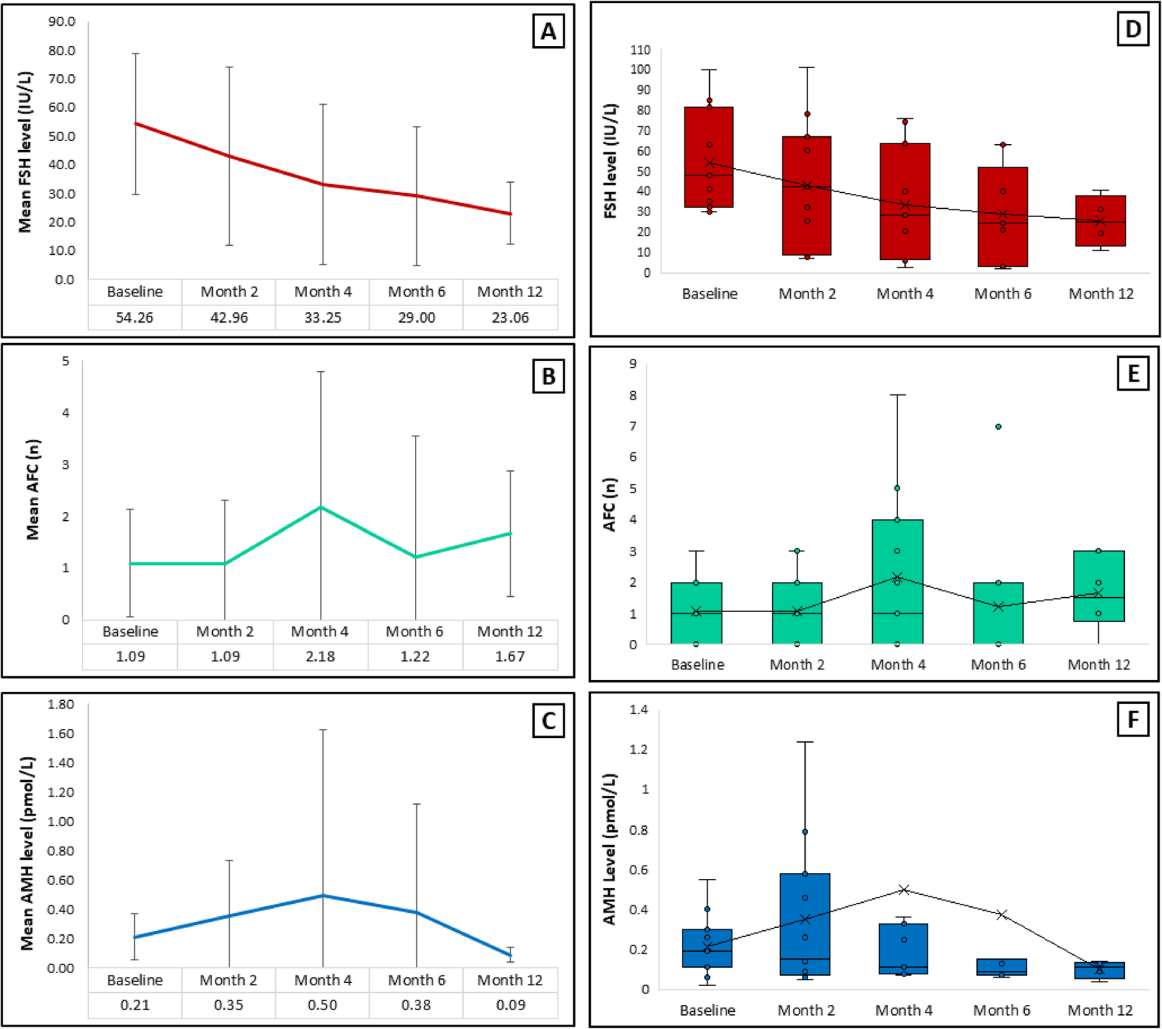
Changes in Ovarian Reserve Markers Following G-CSF Treatment. (**A**–**C**) Line graphs showing mean ± standard deviation of (**A**) serum follicle stimulating hormone (FSH, IU/L), (**B**) antral follicle count (AFC, count), and (**C**) anti-Müllerian hormone (AMH, pmol/L) at baseline and follow-up time points. Numbers are rounded to two decimal places. (**D**–**F**) Boxplots displaying the distribution of individual patient values for (**D**) FSH, (**E**) AFC, and (**F**) AMH across the same time points. Each boxplot depicts the interquartile range, median, mean (×) and mean line, and outliers. Individual patient values are shown as inner dots within each boxplot. One participant exhibited a markedly elevated AMH value at 4 months (3.89 pmol/L). To improve visibility of the remaining data, this outlier is not displayed in panel F; however, it was included in all statistical analyses. A full-scale version including the outlier is provided in Supplementary Figure S1. Statistical analyses were performed using repeated-measures ANOVA with Bonferroni post hoc correction (*p* < 0.05 considered statistically significant). Sample size: n = 11 at baseline through month 4; n = 9 at 6 months and n= 6 at month 12

A repeated-measures ANOVA was conducted to evaluate changes in FSH levels over time at three time points: baseline, 4 months, and 6 months (Table 2). Mauchly’s Test was applied to assess the sphericity assumption. The analysis revealed a significant effect of time on FSH levels, F (2, 16) = 4.684, *p* = 0.025, partial η² = 0.36, indicating that FSH levels decreased significantly across the time points (Fig. 2A and D). Although mean FSH levels decreased significantly from baseline, they remained clinically high, indicating partial rather than full recovery of ovarian function.

**Table 2:** Baseline and follow-up reproductive hormone and ovarian reserve markers in patients receiving treatment. Values are presented as mean ± standard deviation. Comparisons across time points were performed using repeated-measures ANOVA. A *p*-value < 0.05 was considered statistically significant

	Baseline (*n* = 11)	Month 4 (*n* = 11)	Month 6 (*n* = 9)	Month 12 (*n* = 6)	F	*p*-value
FSH (IU/L)	54.26 ± 24.65	33.24 ± 28.01	29.00 ± 24.25	23.05 ± 10.86	8.017	0.002*
AMH (pmol/L)	0.21 ± 0.15	0.49 ± 1.13	0.37 ± 0.73	0.10 ± 0.04	1.328	0.335
AFC (n)	1.09 ± 1.04	2.18 ± 2.60	1.22 ± 2.33	1.66 ± 1.21	0.686	0.574

Post hoc pairwise comparisons, adjusted using the Bonferroni correction, showed a significant reduction in FSH levels at 6 months (29.00 ± 24.25) compared to baseline (*p* = 0.008). However, no significant differences were observed between baseline and 4 months (*p* = 0.181), or between 4 and 6 months (*p* = 1.00), suggesting that the significant decrease in FSH levels occurred specifically between baseline and 6 months.

### AMH levels

Mean AMH levels increased from 0.21 ± 0.15 pmol/L at baseline to 0.49 ± 1.13 pmol/L at 4 months post-treatment (Fig. 2C and F, and Supplementary Fig. 1). Despite this apparent rise, the difference was not statistically significant on a paired sample t-test (t(9) = 0.927, *p* = 0.376).

To assess changes over time, a repeated-measures ANOVA was conducted. Mauchly’s test indicated a violation of the sphericity assumption (*p* = 0.601); therefore, Greenhouse-Geisser correction was applied. The corrected analysis revealed no statistically significant effect of time on AMH levels (F (3,9) = 1.328, *p* = 0.325, partial η² = 0.307), suggesting a small effect size (Table 2). Additionally, AMH concentrations remained close to the assay’s lower limit of detection, reflecting the severely depleted follicular pool in this population.

Bonferroni-adjusted post hoc comparisons further confirmed that changes from baseline to 4 months (*p* = 0.879) and from baseline to 6 months (*p* = 1.000) were not statistically significant.

### AFC

The mean AFC showed a slight increase from baseline to 6-month follow-up (Fig. 2B and E). At baseline, the mean AFC was 1.09 ± 1.04. This increased to 2.18 ± 2.60 at 4 months and declined to 1.22 ± 2.33 at 6 months post-treatment. Despite the observed numerical increase at 4 months, the differences were not statistically significant based on paired sample t-tests (t (9) = 1.636, *p* = 0.133; and t(9) = 0.258, *p* = 0.803, respectively). To be more precise, AFC increased transiently following the third G-CSF cycle, with modest fluctuations observed at subsequent follow-ups, consistent with inter-cycle variability in follicular recruitment.

To evaluate overall change over time, a repeated-measures ANOVA was conducted, which also demonstrated no significant effect of time on AFC (F (3,15) = 0.686, *p* = 0.574, partial η² = 0.121), indicating a small effect size (Table 2). Post hoc comparisons using Bonferroni correction further confirmed the lack of statistically significant differences between baseline and either follow-up (baseline vs. 4 months: *p* = 0.908; baseline vs. 6 months: *p* = 1.000).

These findings suggest that the observed variations in AFC over the study period may be due to random fluctuations rather than an actual treatment effect.

### Menstruation resumption

At baseline, none of the participants reported regular menstrual bleeding; one participant reported irregular bleeding. By 4 months post-treatment, 7 out of 11 participants (63.6%) reported resumption of menstruation. irregular bleeding. By 4 months post-treatment, 7 out of 11 participants (63.6%) reported resumption of cyclic menstruation Menstrual resumption was spontaneous and not related to progesterone withdrawal. In at least 5 of these 7 women, intermittent (untimed) progesterone elevations were measured indicating ovulation. McNemar’s test indicated that this increase was statistically significant (*p* = 0.031), suggesting a meaningful improvement in menstrual activity following treatment. Menstrual resumption was spontaneous and not related to progesterone withdrawal, suggesting potential endogenous follicular activity.

### Individual patient response

Among the 11 patients enrolled, one individual demonstrated a particularly robust clinical and biochemical response following treatment. At baseline, her FSH was 30.1 IU/L, AMH was 0.55 pmol/L, and AFC was 1. By month 4, her FSH had declined to 6.38 IU/L, AMH had increased to 3.89 pmol/L, and AFC rose to 8. She resumed spontaneous menstruation starting the second month post-treatment. Notably, she proceeded to oocyte retrieval, which resulted in the collection of three MII oocytes, representing a significant improvement in ovarian function relative to her baseline status. Because she had no partner, she opted to cryopreserve the 3 mature oocytes. While the case demonstrated notable ovarian recovery, spontaneous remission can occur in a minority of POI patients, and this possibility should be considered when interpreting individual outcomes.

## Discussion

The present study shares conceptual similarities with a previous trial by Herriaz et al. [10], which investigated G-CSF administration for hematopoietic stem cell mobilization in women with DOR. Our study, however, focused specifically on women with premature POI and employed a different dosing regimen including 300 µg of G-CSF administered daily for four consecutive days per month over three months, with follow-up for six months to one year.

Our data confirm that this G-CSF regimen effectively and consistently mobilized hematopoietic stem cells, as demonstrated by significant and reproducible increases in WBC counts following each injection cycle. This hematologic response suggests that the intervention is exhibiting successful biological activity. Despite this, no significant changes were observed in AMH or AFC in 9 of the 11 participants over the 6- to 12-month follow-up period. Serum FSH levels, however, declined significantly, from 54.26 ± 24.65 IU/L at baseline to 33.24 ± 28.01 IU/L at month 4 and 29.0 ± 24.25 IU/L at month 6, suggesting a partial improvement in the gonadotropic hormonal environment.

Of particular interest, although 10 of 11 participants were amenorrheic at study entry (with one reporting irregular menses), seven women resumed menstrual cyclicity following G-CSF treatment. Although AFC and AMH did not show measurable improvement, it is possible that G-CSF–induced hematopoietic stem cell mobilization improved follicular sensitivity to FSH through vascular, immune-modulatory, or paracrine mechanisms. The observed reduction in FSH (while not reaching pre-menopausal levels) may reflect enhanced granulosa cell function with increased inhibin and estrogen secretion, which could be sufficient to restore menstrual bleeding even in the absence of robust changes in ovarian reserve markers. These physiologic improvements may contribute to the possibility of spontaneous conception, despite ovarian parameters remaining suboptimal for IVF. Thus, the clinical utility of G-CSF for assisted reproductive technology in this cohort remains limited. In addition to resumption of menstruation, several patient-reported menopausal symptoms improved following treatment. Statistically significant reductions were observed in vaginal dryness, insomnia, and night sweats, while trends toward improvement were noted for hot flashes, irritability, and poor concentration. These findings suggest that G-CSF treatment may contribute to meaningful improvements in quality of life for patients with POI, even in the absence of robust changes in traditional ovarian reserve markers. Nevertheless, the combination of menstrual recovery and symptom improvement indicates that G-CSF may support ovarian function or hypothalamic-pituitary-ovarian axis activity in a biologically meaningful way, meriting further exploration. Although the small sample size limits statistical power, these results provide preliminary evidence of potential clinical benefit beyond biochemical outcomes.

In contrast to the weak ovarian reserve changes observed in most participants, one patient demonstrated a markedly favourable response, including normalization of FSH, an increase in AFC, resumption of menses, and successful retrieval of three oocytes in an IVF cycle. While this outcome raises the possibility of a true treatment response, spontaneous fluctuations in ovarian function are well-documented in young women with POI. This makes it difficult to attribute such improvement definitively to G-CSF without a control group. Still, her outcome highlights the heterogeneity of POI and suggests that a subset of women might benefit from G-CSF therapy under specific biological conditions.

Various other experimental therapies for POI have been described in the literature with mixed success. One of the first to be published involved in vitro activation by disrupting the Hippo signalling pathway to stimulate dormant primordial follicles [14]. This procedure involved surgical resection of one ovary, mincing the ovarian cortex tissue and treating in vitro with Akt activators. The ovarian tissue fragments were then reimplanted in the pelvis with follicular development in 8/27 patients, and one live baby was born [14]. This procedure involves two surgeries and has not been widely adopted in clinical practice.

Another study in 19 women with POI used laparoscopic ovarian biopsy without chemically induced Akt activation. Resumption of ovarian function occurred in 10 women, and two pregnancies occurred following treatment [15]. Patients with the highest ovarian cortex stiffness were most likely to respond to treatment. The authors speculated that the rigidity of the ovarian cortical extracellular matrix limits expansion of the follicle and oocyte maturation, resulting in quiescent follicles. Reduction of ovarian cortex stiffness by mechanical disruption may be the mechanism of action that allows follicle growth [15].

Another treatment that has been more recently explored is platelet-rich plasma (PRP) injection into the ovaries of women with POI. In one study, 311 women (age 24–40) diagnosed with POI based on ESHRE criteria underwent intraovarian PRP injection [16]. PRP treatment resulted in increased AFC and serum AMH, while serum FSH did not change significantly. After PRP injection, 201 women who developed antral follicles attempted IVF, 82 (26.4% of total) developed embryos, and 57 underwent embryo transfer, resulting in 13 pregnancies (22.8% per transfer, 4% of total). In total, of the 311 women treated with PRP, 25 (8.0%) achieved livebirth/sustained implantation (spontaneously or after IVF), while another 25 (8.0%) had cryopreserved embryos. Recent meta-analyses have further evaluated the efficacy of intraovarian PRP in women with POI and poor ovarian response. One meta-analysis including 23 studies and 1853 participants reported that PRP significantly improved AFC, AMH, and FSH levels in women with POI, and increased the number of retrieved and metaphase II oocytes; pooled pregnancy and live birth rates were 13.8% and 10%, respectively [17]. Another systematic review of 14 studies (1632 participants), including 10 studies on women with POR, one on women with POI, and three on both poor ovarian response and POI, reported significant improvements in AFC, the number of retrieved oocytes, and embryo outcomes in women with poor ovarian reserve, although AMH and AFC changes were not statistically significant [18]. These studies suggest that in women with POI, intraovarian injection of autologous PRP is a potential treatment option for assisted reproductive technology.

The mechanism of action of PRP in POI is unknown. Platelets play a central role in hemostasis. They also contribute to wound healing, mediated by the release of secretory proteins on platelet activation [19]. The α granules of platelets contain numerous proteins that may provide an influence on wound healing, including platelet derived growth factor (PDGF- aa, bb, and ab isomers), transforming growth factor-b (TGF-b, b1 and b2 isomers), platelet factor 4, interleuken-1, platelet-derived angiogenesis factor, vascular endothelial growth factor, epidermal growth factor, platelet- derived endothelial growth factor, epithelial cell growth factor, insulin-like growth factor, osteocalcin, osteonectin, fibrinogen, vitronectin, fibronectin, and thrombospondin-1 [19]. These secretory proteins comprise growth factors, cytokines and chemokines. It is possible that one, or many of these factors together, could contribute to improvement in ovarian function in women with POI. Another speculation is that intraovarian injection of PRP may disrupt the Hippo pathway similar to mechanical disruption of the ovarian cortex with in vitro activation [2]. However, none of the POI treatments, such as surgery to disrupt the Hippo pathway and PRP injections, have undergone controlled studies and pregnancy outcomes with IVF are very poor, less than 5%, consistent with potential spontaneous improvement rather than treatment efficacy.

In the present study of G-CSF injections, aside from the single participant who showed a notable ovarian response, the remaining 10 patients did not demonstrate clinically meaningful improvements in fertility outcomes. Nevertheless, the resumption of menstrual cycles in 7 of the 11 participants, despite persistently low AMH and AFC, is an encouraging observation.

The strength of this study is the enrollment of women with POI, rather than DOR as in most of the previous studies of G-CSF. Patients with POI and menopausal symptoms including elevated FSH are a more homogeneous group and less likely to resolve spontaneously whereas patients with DOR may often show fluctuations in ovarian response from month to month making interpretation of any treatment response less convincing. However, the weakness of our study is the small patient population undergoing treatment. We purposely stopped enrollment in the study since our aim was to achieve an IVF cycle for each patient and the results after 11 patients did not support this aim. It is also possible that there could be some spontaneous fluctuation in ovarian activity even in POI patients which can make interpretation of results problematic in the absence of a control group. Enrollment was intentionally stopped after 11 participants because interim data showed limited ovarian response and the study’s primary objective, to achieve at least one IVF cycle per patient, was not being met. While this decision ensured ethical resource use, it limits the generalizability and statistical strength of the findings. Future studies with larger cohorts and control groups are warranted to confirm these preliminary observations.

In conclusion, this pilot study demonstrates that G-CSF may be biologically active in women with POI, as evidenced by a significant reduction in FSH levels and the resumption of menstruation in over 60% of participants. These findings suggest a potential modulatory effect on the hypothalamic-pituitary-ovarian axis. However, the lack of significant improvements in AMH, AFC, or fertility outcomes limit the strength of the conclusions. Nonetheless, the favourable safety profile and the robust individual response observed in one participant provide a rationale for larger, controlled studies to evaluate efficacy further and to identify subgroups most likely to benefit.

## Supplementary Information

Supplementary Material 1. Supplementary Figure S1. Full-scale boxplot of AMH (pmol/L) including all individual data points (inner dots), showing the outlier value (3.89 pmol/L) at 74 months.

## Data Availability

The datasets used and/or analysed during the current study are available from the corresponding author on reasonable request.

## Abbreviations

AFC: Antral Follicle Count
ALP: Alkaline Phosphatase
ALT: Alanine Aminotransferase
AMH: Anti-Müllerian Hormone
ANOVA: Analysis of Variance
ASCOT: Autologous Stem Cell Ovarian Transplantation
BM: Bone Marrow
BMDSC: Bone Marrow-Derived Stem Cell
BMI: Body Mass Index
CBC: Complete Blood Count
DOR: Diminished Ovarian Reserve
FSH: Follicle-Stimulating Hormone
G-CSF: Granulocyte Colony-Stimulating Factor
IVF: In Vitro Fertilization
LDH: Lactate Dehydrogenase
POI: Premature Ovarian Insufficiency
PRP: platelet-rich plasma
WBC: White Blood Cell (Count)
