# Supplementary material for: Granulocyte colony-stimulating factor treatment in women with premature ovarian insufficiency: a pilot clinical study of biological activity and menstrual resumption
Source: Reprod Biol Endocrinol. 2026 Jan 7;24:19. doi: 10.1186/s12958-025-01510-z (PMC12870933; doi:10.1186/s12958-025-01510-z)
Supplement: Supplementary file 1 — Supplementary Material 1. Supplementary Figure S1. Full-scale boxplot of AMH (pmol/L) including all individual data points (inner dots), showing the outlier value (3.89 pmol/L) at 74 months. [file 12958_2025_1510_MOESM1_ESM.docx]

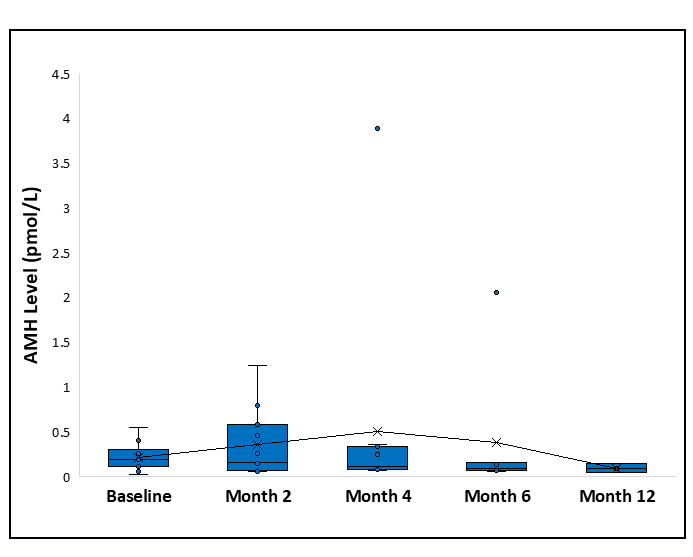


**Supplementary Figure S1.** Full-scale boxplot of AMH (pmol/L) including all individual data points (inner dots), showing the outlier value (3.89 pmol/L) at 74 months.
